# Protective Effects of Inorganic and Organic Selenium on Heat Stress in Bovine Mammary Epithelial Cells

**DOI:** 10.1155/2019/1503478

**Published:** 2019-03-26

**Authors:** Yixuan Zou, Juanjuan Shao, Yongxin Li, F.-Q. Zhao, Jian-Xin Liu, Hongyun Liu

**Affiliations:** ^1^College of Animal Sciences, Zhejiang University, Hangzhou 310058, China; ^2^Department of Animal and Veterinary Sciences, University of Vermont, Burlington, VT 05405, USA

## Abstract

When dairy cows are exposed to high-temperature environment, their antioxidant capacity and productive performance decrease, leading to economic losses. Emerging evidence has shown that selenium (Se) can effectively alleviate heat stress in dairy cows; however, the cellular mechanism underlying this protection is not clear. The purpose of this study was to investigate and compare the protective effects of inorganic Se (sodium selenite, SS) and organic Se (selenite methionine, SM) in MAC-T (mammary alveolar cells-large T antigen, a bovine mammary epithelial cell (BMEC) line) cells during heat stress. MAC-T cells were treated in 4 ways unless otherwise described: (i) cells in the heat treatment (HT) group were cultured at 42.5°C for 1 h and then recovered in 37°C for another 12 h; (ii) the SM group was pretreated with organic Se for 2 h, cultured at 42.5°C for 1 h, and then recovered in 37°C for 12 h; (iii) the SS group was treated similarly to the SM group except that the cells were pretreated with inorganic Se instead of organic Se; and (iv) the control group was continuously cultured in 37°C and received no Se treatment. The results showed that heat shock at 42.5°C for 1 h triggered heat shock response, sabotaged the redox balance, and reduced cell viability in MAC-T cells; and pretreatment of cells with SM or SS effectively alleviated the negative effects of heat shock on the cells. However, the cells were much more sensitive to SS treatment but more tolerant to SM. In addition, two forms of Se appeared to affect the expression of different genes, including nuclear factor erythroid 2-related factor 2 (Nrf2) and inducible nitric oxide synthase (iNOS) in the SM group and thioredoxin reductase 1 (TXNRD1) in the SS group in Nrf2-ARE (antioxidant response element) antioxidant pathway and inflammation response. In summary, results showed the mechanistic differences in the protective effects of organic and inorganic Se on heat stress in BMECs.

## 1. Introduction

As global warming is getting severe, the environmental temperature climbs faster in the recent ten years [1]. Meanwhile, there are over 58% of dairy cows living in the area of torrid and subtropical zones where the temperature humidity index (THI) often reaches over 68 which causes heat stress in dairy cows [2]. Heat stress induces oxidative stress and inflammation, increases the risk of health problems, and reduces milk production [3]. Thus, it is of vital importance to explore effective methods to mitigate the suffering of dairy cows and reduce economic losses in heat stress.

Selenium (Se) is an essential mineral nutrient, and its deficiency in animals is a global problem for susceptibility to various diseases and decreased production performance [4]. In addition, Se can effectively relieve the stress-induced damage in cells. It has been shown that in IPEC-J2 cells, heat stress induced the expression of 10 selenoprotein-related genes which are known to play an important role in antioxidation by promoting the metabolism of hydrogen peroxide and regulating the stress level in cells [5, 6]. In addition, Se can cooperate with immune responses to produce inflammation-related enzymes to kill pathogens [7]. Because of these beneficial effects of Se, various forms of Se have been used as feed additives in animal production in many countries [8]. There are two sources of Se additives, organic and inorganic. Some studies showed that organic Se is less toxic than inorganic selenium [9]. The use of selenized yeast, an organic source of selenium, significantly increases milk selenium concentration compared with inorganic selenium [10], but from the economic point of view, inorganic selenium is more advantageous.

Studies have shown that Se is effective in relieving heat stress in practice [11], but the specific mechanism is still unclear, especially in bovine mammary epithelial cells (BMECs). Therefore, the aim of this study was to determine the function and effects of organic Se (selenite methionine, SM) and inorganic Se (sodium selenite, SS) on antioxidation and anti-inflammation in BMECs.

## 2. Materials and Methods

### 2.1. Cell Culture and Treatment

MAC-T (a BMEC line) cells were cultured in Dulbecco's modified Eagle's medium (DMEM) supplemented with 10% fetal bovine serum (FBS), 100 U/mL penicillin G, and 100 *μ*g/mL streptomycin (Gibco Laboratories, Grand Island, NY, USA) in a humidified incubator at 37°C [12]. The cells were treated in 4 ways unless otherwise described: (i) cells in the heat treatment (HT) group were cultured at 42.5°C for 1 h and then recovered in 37°C for another 12 h; (ii) the SM group was pretreated with various concentrations (0, 0.1, 0.5, 1, 2, 5, 10, 20, 50, and 100 *μ*M) of SM (Sigma, St. Louis, MO, USA) for 2 h, followed by culturing at 42.5°C for 1 h and then recovering in 37°C for 12 h; (iii) the SS group was treated similarly to the SM group except that the cells were pretreated with SS (Sigma) instead of organic Se; and (iv) the control group was continuously cultured in 37°C and received no Se treatment.

### 2.2. Cell Viability Assay

Cell viability assay was performed using the CCK-8 kit (Beyotime, Nanjing, China) according to manufacturer's instruction. MAC-T cells (1 × 10^5^/mL) were seeded into 96-well culture plates. After cells were pretreated with or without different concentrations of SM or SS for 2 h, they were treated or not for 1 h at 42.5°C and then cultured at 37°C for different times. They were then incubated with 10% CCK-8 at 37°C for 2 h before measuring the OD at 450 nm with a microplate reader (MD, CA, USA).

### 2.3. Detection of Intracellular Reactive Oxygen Species

MAC-T cells (1 × 10^6^/mL) after treatment were disposed with 10 *μ*M dichloro-dihydro-fluorescein diacetate (DCFH-DA; Sigma) in 6-well plates at 37°C for 30 min. They were resuspended in phosphate-buffered saline (PBS) and analyzed for fluorescence using flow cytometry. The percentages of fluorescence-positive cells were recorded on a FACSCalibur Flow Cytometer (BD Biosciences, San Diego, CA, USA) using excitation and emission filters of 488 and 530 nm, respectively.

### 2.4. Detection of Apoptosis and Necrosis

Cell apoptosis and necrosis were detected with annexin V-FITC/PI apoptosis detection kit (BD Biosciences). Cells after treatment were harvested, resuspended, and diluted to the density of 1 × 10^6^/mL. After labeling according to manufacturer's protocol, cells were pelleted and analyzed with flow cytometry using excitation filter of 488 nm. The emission filters for green fluorescence of annexin V-FITC and red fluorescence of PI were 525 nm and 595 nm, respectively. Results were analyzed as the percentages of annexin V-FITC^+^/PI^−^ cells by CellQuest software (BD, Franklin Lakes, NJ, USA).

### 2.5. Measurement of Total Antioxidant Capacity and Superoxide Dismutase

Cells were seeded in 6-well culture plates and treated. They were harvested and lysed in ice-cold PBS by sonication, followed by centrifugation at 15,000 g for 10 min at 4°C. The supernatant was taken for subsequent determination. Total antioxidant capacity (T-AOC) was detected using total antioxidant capacity assay kit (Beyotime) with ABTS method [13] following manufacturer's protocol. ABTS stock liquid was prepared for at least 12-16 h before use and stored in no-light condition at room temperature. After diluting to a suitable concentration, 200 *μ*L of ABTS working liquid was added into the supernatant in 96-well culture plates. After mixing and reaction for 2-6 min, the OD of samples was measured at 734 nm with a microplate reader (MD). The T-AOC of the sample was calculated from the standard curve. Superoxide dismutase (SOD) was detected using total superoxide dismutase assay kit (Beyotime) with WST-8 method. Briefly, WST-8/enzyme working liquid and reaction start-up reagent (prepared freshly) were added into 96-well culture plates. After mixing and reaction for 30 min at 37°C, the OD of samples was measured at 450 nm with a microplate reader (MD).

### 2.6. RNA Isolation and Quantitative Real-Time PCR (qPCR)

Total RNA was extracted according to manufacturer's procedures with the RNA Purification Kit (Aidlab Biotechnologies Co. Ltd., Beijing, China). Total RNA of 800 ng was reverse transcribed to cDNA using PrimeScript RT reagent (Takara, Tokyo, Japan) and diluted 1 : 5 for further experiment. QPCR was performed in a 7500c real-time PCR detection system (Applied Biosystems, Carlsbad, California, USA) using SYBR premix EX Taq (Takara) as described previously [14]. GAPDH, RPS9, and UXT were used as housekeeping genes for the normalization of other genes' expression. Primers were designed using the National Center for Biotechnology Information (NCBI) Primer-BLAST and listed in Table 1. The 2^−ΔΔCt^method [15] was used to calculate the relative mRNA abundance.

### 2.7. Western Blotting Analysis

Cells after treatments were lysed on ice by adding 200 *μ*L RIPA buffer containing 10 mM PMSF and scraped into 1.5 mL Eppendorf tubes for centrifugation (12,000 g for 5 min at 4°C). Protein concentrations were determined by BCA protein quantification kit (Beyotime). The lysates were diluted to 2 ng/*μ*L by Sodium Dodecyl Sulfate Polyacrylamide Gel Electrophoresis (SDS-PAGE) loading buffer and separated by SDS-PAGE. Then, proteins were transferred from the gels onto polyvinylidene fluoride (PVDF) membranes (Millipore, USA). The membranes were blocked by 5% milk for 1 h at 4°C under agitation and then incubated with primary antibody against Nrf2 (nuclear factor erythroid 2-related factor 2, 1 : 1000; Abcam, Cambridge, MA, USA), TXNRD1 (thioredoxin reductase 1, 1 : 2000; Abcam), IKB alpha (1 : 1000; Abcam), IKB alpha (phospho S36) (1 : 10000; Abcam), and *β*-actin (1 : 1000; Boster, Wuhan, China) overnight at 4°C. The HRP-conjugated goat anti-rabbit IgG or goat anti-mouse IgG antibodies (Boster) were used as secondary antibodies. The membranes were incubated with secondary antibody for 2 h under agitation. Finally, the western blotting results were quantified using ImagePro Plus 6.0 software (Media Cybernetics, Washington, MD, USA).

### 2.8. Statistical Analysis

Data are presented as mean ± standard deviation of the mean with three independent experiments. Differences between the mean values of normally distributed data were assessed with one-way analysis of variance (ANOVA) followed by Tukey's multiple comparisons test for multiple comparisons and Student's *t*-test for comparisons of two groups. *p* < 0.05 was accepted as statistically significant. All statistical tests were carried out using GraphPad Prism Software version 6.0 (GraphPad Software Inc., La Jolla, CA, USA).

## 3. Results

### 3.1. Se Rescued the Heat Shock-Induced Cell Viability Decrease

**Figure 1:**
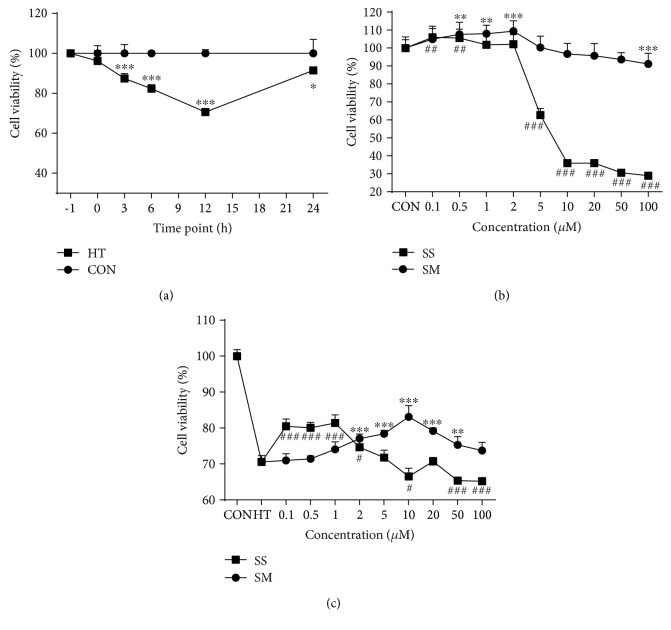
Selenite methionine (SM) and sodium selenite (SS) have protective effects on high-temperature shock-induced cell viability decrease in MAC-T cells. (a) Cell viability of MAC-T cells treated with 42.5°C for 1 h (heat treatment, HT) and then recovered at 37°C for indicated time periods. The control (CON) group was continuously cultured at 37°C without heat treatment. (b) Cell viability of MAC-T cells treated with indicated concentrations of SM and SS for 2 h and then cultured in normal medium for another 12 h. The control group had no SS or SM treatment. (c) Cell viability of MAC-T cells pretreated with indicated concentrations of SM and SS for 2 h, followed by HT and recovery for 12 h. # means significant difference between the SS group and the CON group (b) or between the SS group and the HT group (c). ^∗^ means significant difference between the HT group and the CON group (a), between the SM group and the CON group (b), or between the SM group and the HT group (c). ^#^*p* < 0.05, ^##^*p* < 0.01, ^###^*p* < 0.001; ^∗^*p* < 0.05, ^∗∗^*p* < 0.01, ^∗∗∗^*p* < 0.001.

MAC-T cells treated with heat shock, followed by recovery in 37°C for increasing time periods, showed a gradually decreased cell viability within 12 h (Figure 1(a)). The cell viability was 70% at 12 h of recovery time but nearly fully recovered at 24 h. When the cells were treated with 0.1-100 *μ*M SM or SS for 2 h followed by culturing in normal medium for 12 h, the cell viability increased at low concentrations (0.1-2 *μ*M for SM and 0.1-0.5 *μ*M for SS) but decreased at high concentrations (50-100 *μ*M for SM and 5-100 *μ*M for SS) (Figure 1(b)). Furthermore, when cells were pretreated with 0.1-100 *μ*M SM or SS, followed by heat shock and recovery for 12 h, the heat shock-induced cell viability decrease was partially rescued by 2-100 *μ*M SM or 0.1-2 *μ*M SS pretreatment (Figure 1(c)). Furthermore, the cell viability was further decreased at high concentrations (10-100 *μ*M) of SS. The dose-dependent effects of SM and SS were almost in inverse relationship (Figure 1(c)). Pretreatment of cells with 10 *μ*M SM or 1 *μ*M SS showed the best rescues of cell viability (83.1% and 81.4%, respectively) and thus were used in the following experiments.

### 3.2. Se Alleviated the Heat Shock-Induced Cell Apoptosis and Necrosis

**Figure 2:**
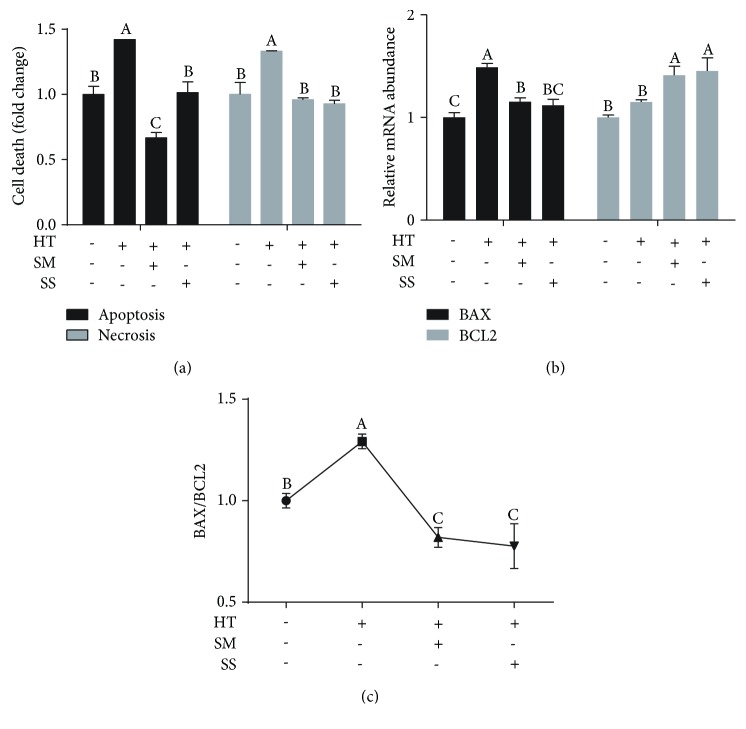
Effects of selenite methionine (SM) and sodium selenite (SS) pretreatments on high-temperature shock-induced cell apoptosis and necrosis (a), mRNA abundance of BAX and BCL2 (b), and the ratio of BAX and BCL2 (c) in MAC-T cells. MAC-T cells were pretreated with (+) or without (-) 10 *μ*M SM or 1 *μ*M SS for 2 h, followed by 42.5°C treatment for 1 h (heat treatment, HT) and recovery at 37°C for 12 h. Values without a common letter are different (*p* < 0.05).

MAC-T cells treated with heat shock increased cell apoptosis and necrosis rates by 1.44- and 1.38-fold, respectively (Figure 2(a)). However, treatment of cells with 10 *μ*M SM or 1 *μ*M SS before heat shock significantly alleviated the heat shock-induced increases in apoptosis and necrosis (Figure 2(a)). In addition, pretreatment with either Se significantly decreased mRNA abundance of BAX (Bcl-2-associated X protein), a proapoptosis marker, and increased mRNA abundance of BCL2 (B-cell lymphoma-2), an antiapoptosis marker (Figure 2(b)), resulting in a decrease in the ratio of BAX and BCL2 in the cells (Figure 2(c)).

### 3.3. Se Reduced Heat Shock-Induced Increase in Heat Shock Response

**Figure 3:**
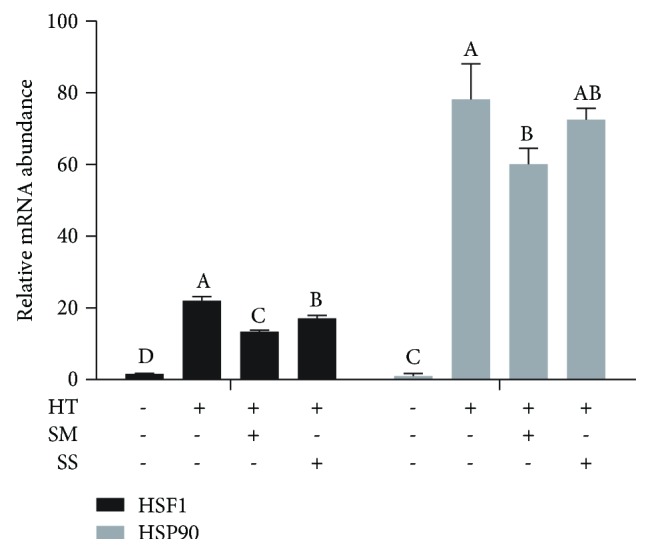
Selenite methionine (SM) and sodium selenite (SS) pretreatments alleviated the increase in mRNA abundance of heat shock factor 1 (HSF1) and heat shock protein 90 (HSP90) induced by high-temperature shock in MAC-T cells. MAC-T cells were pretreated with (+) or without (-) 10 *μ*M SM or 1 *μ*M SS for 2 h, followed by 42.5°C treatment for 1 h (heat treatment, HT) and recovery at 37°C for 12 h. Values without a common letter are different (*p* < 0.05).

Heat shock response (HSR) was triggered by high-temperature treatment in MAC-T cells as shown by the large increase in mRNA abundance of heat shock factor 1 (HSF1) and heat shock protein 90 (HSP90) in Figure 3. The response of HSF1 was reduced by the pretreatment of cells with 10 *μ*M SM or 1 *μ*M SS, whereas the HSP90 increase was decreased by pretreating cells with SM only (Figure 3).

### 3.4. Se Improved the Heat Shock-Induced Decline in Antioxidant Capacity

**Figure 4:**
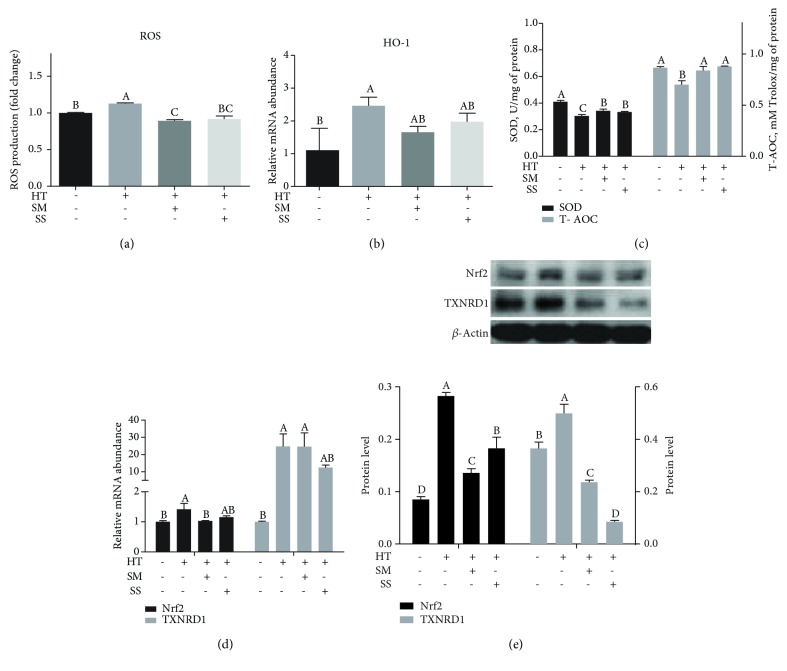
Selenite methionine (SM) and sodium selenite (SS) improved redox status in high temperature-shocked MAC-T cells. MAC-T cells were pretreated with (+) or without (-) 10 *μ*M SM or 1 *μ*M SS for 2 h, followed by 42.5°C treatment for 1 h (heat treatment, HT) and recovery at 37°C for 12 h. The cells were analyzed for (a) the intracellular reactive oxygen species (ROS) production; (b) relative mRNA abundance of HO-1; (c) the superoxide dismutase (SOD) activity and total antioxidant capacity (T-AOC); (d) relative mRNA abundance of nuclear factor erythroid 2-related factor 2 (Nrf2) and its target gene thioredoxin reductase 1 (TXNRD1); and (e) protein levels of Nrf2 and TXNRD1 (top panel: a representative western blot image, bottom panel: quantitative representation of the western blot analysis of 3 independent experiments). Values without a common letter are different (*p* < 0.05).

As shown in Figure 4(a), the level of ROS (reactive oxygen species) increased after heat shock in MAC-T cells, and the pretreatment of cells with 10 *μ*M SM or 1 *μ*M SS significantly reduced the ROS increase in heat shock-treated cells. In addition, the mRNA abundance of HO-1 (heme oxygenase 1) was increased in the HT group compared with the control group, but the increase was partially inhibited by SM and SS pretreatments (Figure 4(b)). The mRNA abundance of SOD and T-AOC were decreased when cells were treated with high temperature, and SM and SS pretreatments improved these declines (Figure 4(c)). Furthermore, the protein levels of Nrf2 and TXNRD1 were increased by heat shock treatment, but the increase was suppressed by SM or SS pretreatment (Figure 4(e)). It appeared that the effect of SM pretreatment was more on Nrf2 expression, whereas the effect of SS tended to be more on inhibiting the protein level of TXNRD1 (Figures 4(d) and 4(e)).

### 3.5. Se Reduced the Influence of Heat Shock on Inflammation

**Figure 5:**
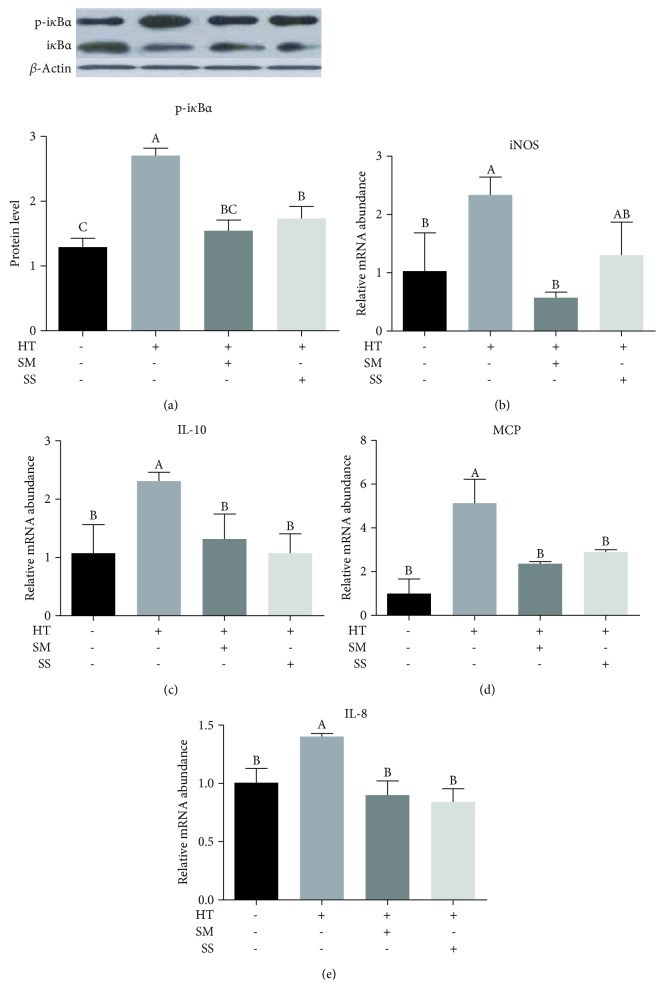
Selenite methionine (SM) and sodium selenite (SS) alleviated the inflammation responses induced by high-temperature shock in MAC-T cells. MAC-T cells were pretreated with (+) or without (-) 10 *μ*M SM or 1 *μ*M SS for 2 h, followed by 42.5°C treatment for 1 h (heat treatment, HT) and recovery at 37°C for 12 h. The cells were analyzed for the protein level of i*κ*B*α* ((a) top panel: a representative western blot image, bottom panel: quantitative representation of the western blot analysis of 3 independent experiments) and mRNA abundance of inducible nitric oxide synthase (iNOS) (b), interleukin-10 (IL-10) (c), monocyte chemoattractant protein (MCP) (d), and interleukin-8 (IL-8) (e). Values without a common letter are different (*p* < 0.05).

The protein level of i*κ*B*α* (Figure 5(a)) and the mRNA abundance of inflammatory cytokines and markers, such as inducible nitric oxide synthase (iNOS; Figure 5(b)), interleukin-10 (IL-10; Figure 5(c)), monocyte chemoattractant protein (MCP; Figure 5(d)), and interleukin-8 (IL-8; Figure 5(e)), were all increased by heat shock in MAC-T cells (Figure 5). However, these increases were mostly reversed by SM or SS pretreatment. Specifically, SM suppressed mRNA abundance of iNOS significantly (Figure 5(b)), whereas SS did not show a significant effect.

## 4. Discussion

Elevated environmental temperature changes not only the behaviors of dairy cows, such as malaise, panting, frustration, and aggression, but also the biological function and health status which cause milk yield decrease and delay in estrus cycles [16]. But the specific mechanism of heat stress in cows is still unclear. Our study showed that heat shock treatment of BMECs with 42.5°C for 1 h reduced cell viability at 12 h by 30%, and this reduction was at least partially caused by increasing cell apoptosis and necrosis rates. The ratio of BAX/BCL2 in these cells also confirmed the effect. This observation was consistent with an earlier study [17].

The dilemma that high-producing cows are more susceptible to environmental temperatures makes it a high priority to find effective methods to reduce heat stress in cows [18]. Although fans, sprinkles, misters, and cooled waterbeds have been used in dairy farms to alleviate heat stress, researchers working on the effect of heat stress on dairy cows have increasingly focused on developing methods to use supplemental dietary additives, for example, Se [19, 20], to alleviate the hyperthermia damages in cows. Se has the protective function against oxidative stress in dairy cows [21]. As a common antioxidant trace element, Se also has anti-inflammatory, anticancer, and immune-enhancing functions [22]. The addition of Se has shown to increase the resistance of the breast to inflammatory diseases [23]. Se includes organic and inorganic forms. To our knowledge, until now there are not any studies to investigate their differences in the molecular mechanism in resisting heat stress.

The present study found that pretreatment of MAC-T cells with SM or SS itself had significant effect on cell viability in a dose-dependent manner. MAC-T cells were much more sensitive to the concentrations of SS compared to SM. Some studies showed that organic Se has higher bioavailability and is more environmental friendly and less toxic to animals than inorganic Se [9, 24, 25]. A study showed that cattle with organic Se supplementation had higher concentrations of Se in whole blood and milk than cattle with inorganic Se supplemented [10]. The difference may be due to the distinct absorption mechanism of two forms of Se from the gastrointestine. Kim and Mahan [9] showed that dietary organic and inorganic Se were toxic when the concentrations of Se in growing-finishing swine exceeded 5 ppm, but subsequent selenosis was worse and appeared earlier if SS was used as Se source. Consistently, in this study, pretreatment of cells with SM or SS also partially rescued the decrease in cell viability of MAC-T after 42.5°C shock for 1 h in a dose-dependent manner. The cell viability after heat shock significantly improved at low concentrations of SS (0.1-2 *μ*M) but at higher concentrations of SM (≥2 *μ*M).

Studies have shown that cells have increased susceptibility to undergoing apoptosis when suffering heat stress [26]. In this study, the improved cell viability after heat shock by SM and SS pretreatments was also supported by the decrease in cell apoptosis and necrosis rates and decrease in the ratio of BAX/BCL2, two major cell apoptosis-associated regulatory proteins. The protective effect of Se on heat stress in MAC-T cells is consistent with the results of the studies of Khera et al. [27] in trophoblast cells and Ganesan et al. [28] in muscle cells.

The main function of HSP (a heat shock protein) is to resist the effects of stress on cells [29]. HSF1 leads the induction of expression of stress-responsive genes, whereas HSP90 is a main defense protein against heat stress [30, 31]. Under normal circumstances, HSF1 binds with HSP (usually HSP90). When the body or cells are stimulated, HSP is separated from HSF1. HSF1 then enters the nucleus and induces the expression of downstream heat shock element regulatory genes [1]. Our study showed that there was a striking increase of gene expression of HSF1 and HSP90 in MAC-T cells after heat shock and Se pretreatment reversed the effects. The findings are supported by previous observations that Se deficiency increased the level of heat shock proteins in chicken livers [32] and the expression of HSP90 in chicken erythrocytes [33].

Studies have discovered that heat stress can enhance the production of ROS and then disturb the homeostasis of redox equilibrium, leading to oxidative stress in cells [34]. In addition, heat stress changed the expression of selenoprotein genes in IPEC-J2 cells [5], which could further contribute to heat stress in the cells. Study also found that the supplementation of Se can increase the glutathione peroxidase activity and improve the ability of antioxidant system in lactating cows [35]. Our study confirmed that oxidative stress in MAC-T cells after heat shock increased ROS production and decreased SOD and T-AOC activity. Our study also showed the antioxidative effect of Se pretreatment in MAC-T cells. The antioxidative effect of Se is likely due to its existence in redox system-related enzymes, such as glutathione peroxidase (GSH-Px). Furthermore, heat shock and Se pretreatment influenced the expression of HO-1. Endogenous carbon monoxide generated by HO-1 activates Akt/PKB (protein kinase B). Akt has a negative impact on GSK-3*β* (glycogen synthase kinase 3*β*), which activates Nrf2 [36]. Thus, the effects of Se can also result from its effect on the expression of Nrf2, a master transcription factor that regulates the expression of antioxidant proteins. A previous study showed that the influence of epigallocatechin-3-gallate on the intracellular Nrf2 levels was removed in Se-optimal mice [37]. Upon oxidative stress, the ubiquitination of Nrf2 stops. Nrf2 translocates into the nucleus and binds with antioxidant response element, ultimately activating the defensive system [38]. TXNRD1, an intracellular selenoprotein, is an isozyme which provides one of the main enzymatic defense systems for ROS in vascular endothelial cells [39]. In this study, we found that two forms of Se tended to activate different genes in Nrf2-antioxidant pathway. SM pretreatment tended to suppress the expression of Nrf2, whereas SS tended to decrease the protein level of TXNRD1.

Oxidative stress has been linked to inflammation [40]. The appearance of inflammation is usually mediated by cytokines. IL-10 has a pleiotropic effect in immune regulation and inflammation. It blocks the activity of NF-*κ*B (nuclear factor kappa-B) and participates in the regulation of JAK-STAT (Janus kinase-signal transducer and activator of transcription) signaling pathway [41]. A large amount of NO (nitric oxide) is produced by iNOS during stimulation. The induction of high output of iNOS usually occurs in oxidizing environment so that high levels of NO have the opportunity to react with superoxide, leading to peroxynitrite formation and cytotoxicity [42]. In this study, we showed that heat shock induced inflammation as shown by increased mRNA abundance of inflammation markers and cytokines, and Se pretreatment can suppress the induction. SM pretreatment regulated iNOS prominently, whereas SS had no significant effect on iNOS.

## 5. Conclusion

SM and SS are two Se dietary additives commonly used in dairy farms. However, their concentrations and effects on milk production are diverse from each other. The present study showed that both SM and SS can relieve heat stress damage in MAC-T cells. Specifically, SM and SS can modulate the antioxidant and immune responses through different enzymes and cytokines. The organic Se showed more effects on an upper stream target (Nrf2) on oxidative stress and iNOS on inflammation, whereas inorganic Se acted more on the lower stream target (TXNRD1). This study showed the different target genes on oxidative stress and inflammation by two forms of Se on cellular level for the first time.

## Figures and Tables

**Table 1:** Sequences of primers used in real-time PCR.

Gene	Forward (5′-3′)	Reverse (5′-3′)	GenBank accession # of mRNA
RPS9	cctcgaccaagagctgaag	cctccagacctcacgtttgttc	NM_001101152.2
UXT	tgtggcccttggatatggtt	ggttgtcgctgagctctgtg	NM_001037471.2
GAPDH	tggaaaggccatcaccatct	cccacttgatgttggcag	NM_001034034.2
BAX	tggacattggacttccttcg	ccagccacaaagatggtcac	NM_173894.1
BCL2	ggggtcatgtgtgtggagag	tccacaaaggcgtcccag	NM_001166486
HSF1	tgcagctgatgaaggggaag	actggatgagcttgttgacga	NM_001076809.1
HSP90	ccaagtctggcactaaag	gaagactcccaagcatac	NM_001079637.1
Nrf2	aaccaccctgaaagcacaac	ttgggacccttctgtttgac	NM_001011678.2
TXNRD1	gtgttcacgactctgtcggt	ctgccttccacgaatcacct	NM_174625.4
HO-1	atcgaccccacacctacaca	gacgccatcaccagcttaaaa	NM_001014912
iNOS	tcaacaaagccctgagcagta	ggaaaactccgaggtgctct	NM_001076799.1
MCP	cgctcagccagatgcaatta	cccatttctgcttggggtct	NM_174006.2
IL-8	ttgtgaagagagctgagaagca	acccacacagaacatgaggc	NM_173925.2
IL-10	ctttaagggttacctgggttgc	gccttgctcttgttttcgca	NM_174088.1

## Data Availability

The data used to support the findings of this study are available from the corresponding author upon request.
